# An *in vitro* reproduction of stress-induced memory defects: Effects of corticoids on dendritic spine dynamics

**DOI:** 10.1038/srep19287

**Published:** 2016-01-14

**Authors:** Shinichi Saito, Satoshi Kimura, Naoki Adachi, Tadahiro Numakawa, Akihiko Ogura, Keiko Tominaga-Yoshino

**Affiliations:** 1 Laboratory of Synaptic Plasticity, Osaka University Graduate School of Frontier Biosciences, Suita, Osaka 565-0871 Japan; 2 National Center of Neurology and Psychiatry, Kodaira 187-8551 Tokyo, Japan

## Abstract

Previously, in organotypic slice culture of rodent hippocampus we found that three repeated inductions of LTP, but not a single induction, led to a slow-developing long-lasting enhancement of synaptic strength coupled with synapse formation. Naming this structural plasticity RISE (repetitive LTP-induced synaptic enhancement) and assuming it to be a potential *in vitro* reproduction of repetition-dependent memory consolidation, we are analyzing its cellular mechanisms. Here, we applied a glucocorticoid to the culture to mimic acute excess stress and demonstrated its blockade of RISE. Since excess stress interferes with behavioral memory consolidation, the parallelism between RISE *in vitro* and memory consolidation *in vivo* is supported. We recently reported that RISE developed after stochastic processes. Here we found that the glucocorticoid interfered with RISE by suppressing the increment of dendritic spine fluctuation that precedes a net increase in spine density. The present study provides clues for understanding the mechanism of stress-induced memory defects.

From daily experience, we are aware that memory is solidified and becomes long lasting through repetition. Historically, Ebbinghaus showed that repeated task performance decelerated the ‘forgetting curve’ for a random list of meaningless words^1^. This is also true in animal experiments; standard protocols apply repeated training to establish learning^2^, not to mention the classical conditioning studies by Pavlov^3^. However, the cellular bases underlying this repetition-dependent memory consolidation remain largely unknown. For example, it is unclear whether each single experience produces identical signals in the cell, which are then accumulated stepwise, or if the first and subsequent experiences produce different signals. One of the reasons for these uncertainties is the lack of appropriate *in vitro* model systems that allow for an experimenters’ control.

By using the stable organotypic slice culture of rodent hippocampus, we reported previously that repeated inductions, but not a single induction, of long-term potentiation (LTP) produced a slow-developing (requiring approximately 1 week for full development) and long-lasting (lasting for more than 2 weeks) enhancement of synaptic strength in the CA3-CA1 synapses, accompanied by new synapse formation^4^,^5^. This structural plasticity phenomenon, named RISE (repetitive LTP-induced synaptic enhancement), shows parallelism with behavioral memory consolidation in various aspects, including not only the requirement of repetition and longevity but dependency on protein synthesis^6^ and input pathway specificity^7^.

However, evidence for a treatment that interferes with memory consolidation *in vivo* as well as with RISE *in vitro* is still lacking. In the present study, we examine whether acute excess stress known to cause memory defects *in vivo*^8^,^9^ also interferes with RISE *in vitro* by simulating excess stress conditions with the application of glucocorticoids to the culture^10^,^11^.

When the parallelism between memory consolidation *in vivo* and RISE *in vitro* is established, we may then proceed to the next step: to obtain evidence for which cellular process stress does interfere with. In other words, RISE may serve as a model system for analyzing the effect of stress on the cellular basis, freed from the problems of complex homeostatic feedback responses that occur when using a whole animal model.

We recently reported that RISE developed stochastically following four consecutive phases: 1) dendritic spines (postsynaptic structure) are under constant dynamic equilibrium between generation and retraction; 2) a RISE-producing stimulus transiently enhances both rates of generation and retraction; 3) the retraction rate returns to the pre-stimulus level before the generation rate, resulting in a net increase in spine density; and 4) the generation rate returns to the pre-stimulus level, re-establishing the dynamic equilibrium^12^. In principle, suppressing either of these phases will result in the suppression of spine density increase. The question is which phase is actually affected ? The answer will provide new information for understanding the cellular basis of stress-induced memory defects.

## Results

### Dexamethasone suppresses establishment of RISE

In *ex vivo* studies, exogenous applications of glucocorticoids are often employed to simulate excess stress conditions^8^. The major glucocorticoid species working in the rodent is corticosterone. However, here we used dexamethasone (Dex) as a glucocorticoid receptor agonist to minimize the untoward effects of corticosterone on mineralocorticoid receptors.

**Figure 1:**
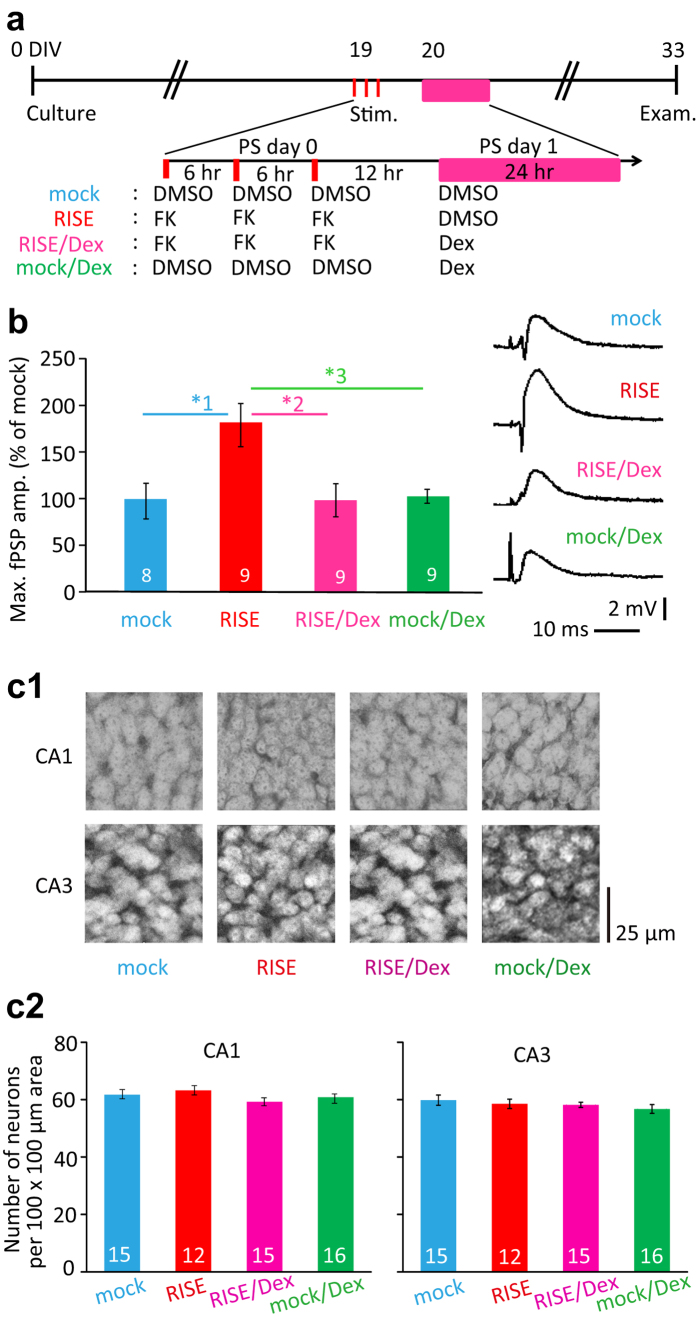
Suppression of RISE in cultured hippocampal slices by Dex, applied 12 hr after stimulation. (**a**) Experimental timeline for electrophysiological examination. Culture age was counted as the day of culture 0 DIV (days *in vitro*). At 19 DIV, FK (forskolin; 20 μM, 20 min) or DMSO (dimethylsulfoxide; vehicle for FK) was applied three times at 6 hr intervals. This day was reckoned as PS (post-stimulus) day 0. Dex (100 nM) was applied for 24 hr beginning 12 hr after the third application of FK (*i.e.* PS day 1). At PS day 14 (*i.e.* 33 DIV), electrophysiological examinations were performed. (**b**) Maximal fPSP amplitude taken as a functional index for RISE. As explained in Methods section, the control (mock) and experimental samples were subjected to fPSP measurements on the same day using a single lot of culture. The mean value of control samples was taken as 100% for each lot. In this and following figures, the numeral placed in each bar of the graph indicates the number of samples constructing the data shown. The same measurement was repeated 4 times using 4 lots of culture (*i.e.* 4 dams). Absolute *P* values are 2.15E-2 for *1, 1.50E-2 for *2, 2.42E-2 for *3. (**c**) Absence of cytotoxic effect of Dex. Representative fluoromicrographs are shown in c1 and pooled data are shown in c2. The numbers of NeuN-immunopositive nuclei were enumerated for ~50 × ~50 μm areas of the CA1 and CA3 pyramidal layers at PS day 14 and shown after conversion to 100 × 100 μm areas. The same measurement was repeated 4 times using 4 lots of culture (*i.e.* 4 dams).

Glucocorticoids are known to suppress the induction of LTP^13^,^14^. Since RISE develops after repeated LTP inductions, it is useless to apply Dex before or during cLTP induction to know the possible glucocorticoid’s effect to RISE. Hence, we applied Dex to the culture for 24 hr beginning 12 hr after the third cLTP induction (see Fig. 1a for experimental timeline).

As shown in Fig. 1b, three repeated inductions of cLTP produced RISE, when fPSP was assayed at post-stimulus (PS) day 14. However, Dex applied 12 hr after the third cLTP suppressed the establishment of RISE.

Dex at this dose and duration of application did not cause neuronal cell death in the areas CA1 and CA3; the possibility that cell death cancelled the synaptic enhancement has been eliminated (Fig. 1c).

**Figure 2:**
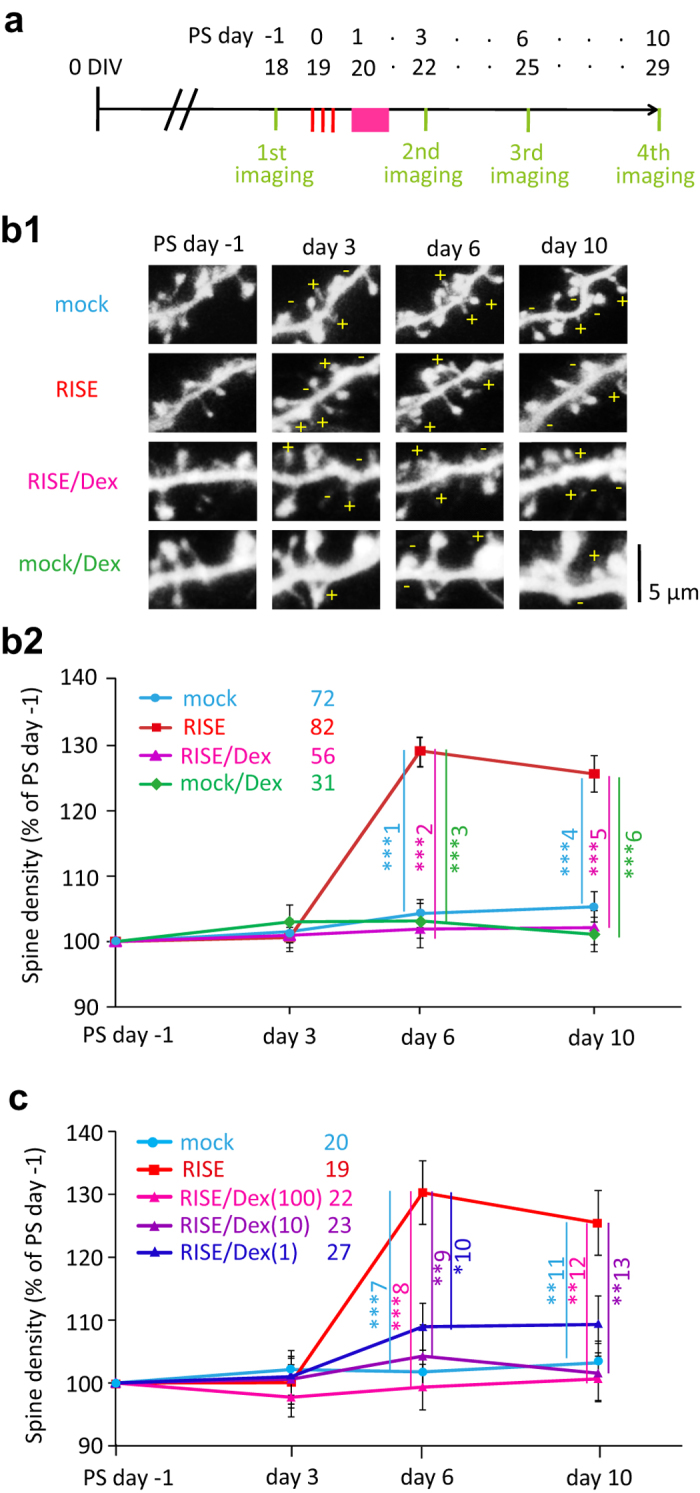
Dose-effect relationship of Dex on RISE suppression. (**a**) Experimental timeline for morphological examination. The same dendritic segments were imaged periodically at PS day −1, 3, 6, and 10. (**b**) Spine density taken as a morphological index for RISE. Representative images are shown in b1 and pooled data are shown in b2. The number of spines was counted along a >10 μm length of dendritic segment and that at PS day −1 was taken as 100% for this segment. As explained in Methods section, the control (mock) and experimental samples were subjected to spine density measurements on the same day using a single lot of culture. The mean value of control samples was taken as 100% for each lot. The same measurement was repeated 12 times using 12 lots of culture (*i.e.* 12 dams). The number of segments examined to construct each curve of the chart is indicated in the note in the panel. Absolute *P* values are 6.28E-13 for ***1, 2.32E-13 for ***2, 6.62E-9 for ***3, 1.20E-8 for ***4, 9.93E-10 for ***5, 1.68E-7 for ***6. (**c**) Dose-effect relationship of Dex on RISE suppression. This experiment was performed separately from that depicted in b using separate lots of culture, so that the absolute values (*e.g.* of those for RISE and RISE/Dex (100 nM)) are slightly different. The same measurement was repeated 4 times using 4 lots of culture (*i.e.* 4 dams). Absolute *P* values are 1.19E-4 for ***7, 1.49E-5 for ***8, 3.33E-4 for ***9, 3.70E-3 for **10, 9.48E-3 for **11, 1.84E-3 for **12. 2.66E-3 for **13. For the functional index (fPSP at PS day 14), see Supplementary Fig. S2.

As a morphological index, we measured the number of dendritic spines (see Fig. 2a for experimental timeline). Dex suppressed the increase in spine number (Fig. 2b). It is noteworthy that Dex did not decrease the spine number (the fPSP amplitude as well) by itself, meaning that the observed suppression of RISE was not a mixture of the spine-incremental effect of RISE and spine-decremental effect of Dex.

When the spine number was counted at PS day 10, the suppressive effect of Dex was apparent at 10 nM (Fig. 2c). Dex of 1 nM also showed a suppressive effect, but the effect was smaller. Since the culture medium should have contained ≤0.3 nM glucocorticoid (cortisol) originating from the horse serum (see Methods), the exogenously applied 1 nM Dex might have been non-salient.

The RISE-suppressive tendency of 1 nM Dex was also seen in the functional index (fPSP amplitude) (Supplementary Fig. S1).

### Mineralocorticoid has no suppressive effect on RISE

**Figure 3:**
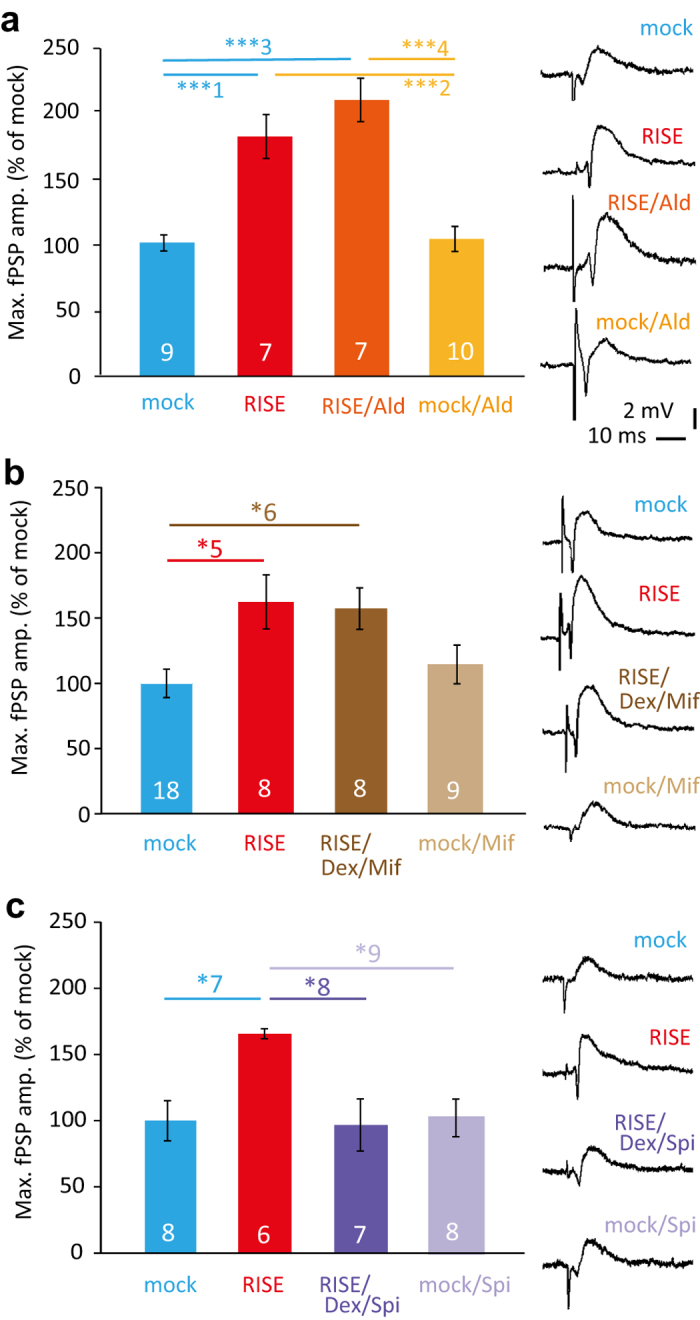
RISE suppressive effect of corticoid is mediated by the glucocorticoid pathway. The functional index (fPSP at PS day 14) is depicted here. (**a**) Absence of effect of a mineralocorticoid receptor agonist aldosterone (Ald; 10 nM). The same measurement was repeated 4 times using 4 lots of culture (*i.e.* 4 dams). Absolute *P* values are 3.98E-4 for ***1, 4.56E-4 for ***2, 4.68E-6 for ***3, 4.85E-6 for ***4. For the morphological index (spine density), see Supplementary Fig. S2. (**b**) Cancellation of RISE suppressive effect of Dex by a glucocorticoid receptor antagonist mifepristone (Mif; 0.5 μM). The same measurement was repeated 4 times using 4 lots of culture (*i.e.* 4 dams). Absolute *P* values are 2.42E-2 for *5, 4.67E-2 for *6. (**c**) No cancellation of RISE suppressive effect of Dex by a mineralocorticoid receptor antagonist spironolactone (Spi, 0.5 μM). The same measurement was repeated 3 times using 3 lots of culture (*i.e.* 3 dams). Absolute *P* values are 3.60E-2 for *7, 3.15E-2 for *8, 4.53E-2 for *9.

The literature demonstrates that mineralocorticoids also interfere with LTP^14^. Hence, it is possible that mineralocorticoids would also affect RISE. We applied aldosterone (Ald; 10 nM) according to the same protocol as Dex, and found the establishment of RISE was not suppressed (or enhanced) either in fPSP amplitude at PS day 14 (Fig. 3a) or in the spine density at PS day 10 (Supplementary Fig S2). Ald, by itself, showed no effect to RISE in either functional or morphological indices.

These results indicate that that Dex interferes with RISE through the glucocorticoid receptor. This notion was further confirmed by the effect of the glucocorticoid receptor antagonist mifepristone (Mif), which cancelled the RISE-suppressive effect of Dex (Fig. 3b; see RISE/Dex/Mif), and by the absence of cancellation of the Dex’s RISE-suppressive effect upon the addition of the mineralocorticoid receptor antagonist spironolactone (Spi) (Fig. 3c; see RISE/Dex/Spi).

It is possible that a low dose glucocorticoid contaminating in the serum may have influenced the basal synaptic activity through corticoid receptors, since glucocorticoids can activate both glucocorticoid and mineralocorticoid receptors. However, this possibility is low, since Mif and Spi did not alter the basal fPSP (Fig. 3b,c; see mock/Mif and mock/Spi, respectively).

### Dexamethasone suppresses the stochastic processes leading to RISE

The results shown above support our hypothesis that RISE is an *in vitro* reproduction of repetition-dependent memory consolidation process *in vivo* by adding a novel component to the list of parallelisms between RISE and repetition-dependent memory consolidation. The benefit of RISE as a model system for analyzing the cellular bases of memory consolidation lies in its ability to be utilized in the analyses of stress-induced memory defects.

As explained in the introductory section, RISE develops according to stochastic structural changes that include 4 consecutive phases^12^: 1) the dynamic equilibrium between spine generation and retraction (balanced fluctuation phase); 2) transient increase in the both rate of spine generation and retraction (increased fluctuation phase); 3) the rate of retraction returns to the pre-stimulus level before that of the rate of generation (biased fluctuation phase); 4) the rate of generation returns to the pre-stimulus level (re-balanced fluctuation phase). We addressed the question on which phase Dex affects to lead to the suppression of the spine number increase.

**Figure 4:**
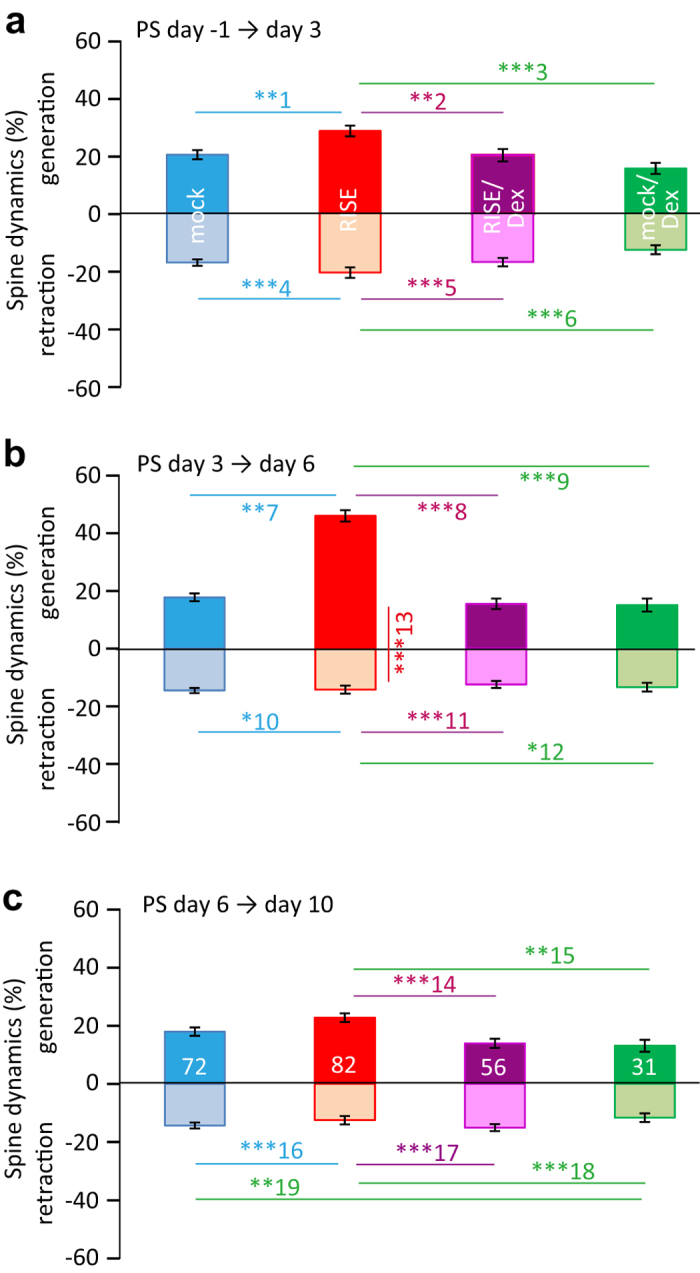
Dendritic spine dynamics in RISE and interference by Dex. The same dendritic segments (of >10 μm in length) were imaged and comparisons were made between two consecutive images (as exemplified in Fig. 2b1). Spines absent in the first image but present in the second were classified as ‘generated’ spines. Those present in the first image but absent in the second were classified as ‘retracted’ spines. Spines displaced laterally within 0.7 μm were judged identical. The proportions of the number of generated and retracted spines to the number of spines present in the first image were plotted. Generation is shown in the plus direction and retraction in the minus direction of the ordinate. Since identical dendritic segments were chased, the sample numbers are common throughout and shown in the bottom panel. The same spine fate chasing was repeated 12 times using 12 lots of culture (*i.e.* 12 dams). Absolute *P* values are 5.94E-3 for **1, 7.90E-3 for **2, 4.50E-5 for ***3, 4.15E-5 for ***4, 1.16E-4 for ***5, 1.02E-6 for ***6, 3.51E-25 for ***7, 2.60E-24 for ***8, 4.60E-19 for ***9, 1.61E-2 for *10, 4.22E-4 for ***11, 2.60E-2 for *12, 3.51E-22 (Welch’s *t*-test) for ***13, 9.48E-4 for ***14, 1.99E-3 for **15, 2.20E-4 for ***16, 2.78E-7 for ***17, 3.15E-9 for ***18, 9.46E-3 for **18.

Figure 4 shows apparent rates of spine generation and retraction estimated by microscopic examinations performed at 3–4 day intervals. We call these rates ‘apparent’ since the true rates should be higher, because spines generated and quickly retracted within an examination interval were not captured in this study.

Figure 4a shows the initial ‘increased fluctuation’ phase. The RISE-stimulated cultures showed an elevated fluctuation (*i.e.* increments in both rate of spine generation and retraction). Dex suppressed this elevated fluctuation (see RISE versus RISE/Dex). It should be noted that Dex did not influence the basal fluctuation (see mock versus mock/Dex) significantly.

Figure 4b shows the next ‘biased fluctuation phase’. In the RISE-stimulated cultures, the rate of spine generation was larger than than that of retraction, leading to a net increase in spine number. The Dex-applied cultures did not enter in this phase.

Figure 4c shows the final ‘re-balance fluctuation phase’ where the increased rate of spine generation in RISE-stimulated cultures returns to the basal level. The RISE-stimulated cultures still showed a slightly higher rate of spine generation when compared with the Dex-treated cultures, presumably because the examination period here (between PS day 6 and 10) might be slightly early. The RISE-stimulated cultures show a small but significant ‘undershoot’ in the rate of retraction, which was also seen in previous examination (Fig. 7 of ref. 12).

In summary, Dex suppressed the rate of spine generation from the phase 2 onward. In a separate series of experiments, an absence of effect of exogenously applied Ald was confirmed (Supplementary Fig. S2).

### RISE-suppressive effect of dexamethasone has an effective time frame

**Figure 5:**
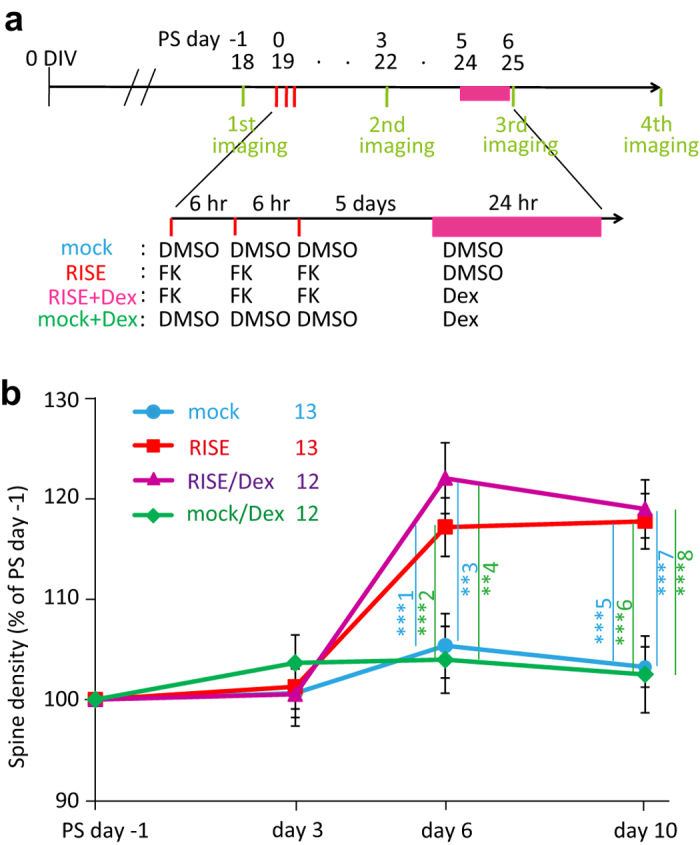
Time frame sensitivity of RISE to Dex. The application of Dex was shifted to PS day 5. The morphological index of RISE is depicted. Note that RISE is produced uninterruptedly. (**a**) Experimental timeline for morphological examination. (**b**) Time course of spine density changes. The same measurement was repeated 2 times using 2 lots of culture (*i.e.* 2 dams). Absolute *P* values are 2.34E-2 for *1, 3.00E-2 for *2, 1.11E-3 for **3, 1.56E-3 for **4, 4.58E-3 for **5, 3.32E-3 for **6, 2.29E-3 for **7, 1.63E-3 for **8.

When we delayed the application of Dex to PS day 5 (instead of PS day 1 in the above experiments), the RISE-suppressive effect was not apparent when assayed morphologically at PS day 10 (Fig. 5). This result may mean that the glucocorticoid interferes with the initiation of RISE but not its maintenance.

## Discussion

Excess stress is known to suppress memory consolidation in both animals and humans^8^,^9^. The *in vitro* phenomenon of repeated inductions of LTP resulting in a long-lasting synaptic strengthening, coupled with structural changes^4^, called RISE, is suppressed by the application of glucocorticoid, which simulates the excess stress conditions *in vivo*^10^. The parallelism between RISE *in vitro* and repetition-dependent memory consolidation *in vivo* shown here, together with the previously reported parallelisms, supports our aim that the cellular bases for repetition-dependent memory consolidation should be analyzed using this phenomenon as a model system.

Animals have robust homeostatic mechanisms^15^, including the hypothalamus-pituitary-adrenal gland (HPA) axis and additional local feedback loops that counteract to treatments intended by the experimenter; these complex animal systems may confound the analysis of the effects of stress. It is therefore desirable to establish an *in vitro* model system that allows direct manipulation of neuronal environments free from the complex feedback systems of a whole animal. We propose that RISE may serve as a useful model system.

One of the promising directions using this system is the interference of stress with BDNF (brain-derived neurotrophic factor) signaling. There are many reports that describe the influence of stress to the expression and action of BDNF. But the results are intricate; both up- and downregulation are reported (reviewed by ref. 16). Presumably the direct and indirect (*i. e.* resulted from the homeostatic action of HPA axis) effects of stress would be mixed. The establishment of RISE depends on BDNF signaling^17^. Then, the cellular mechanisms underlying the stress-induced memory defects may be analyzed by pursuing the direct action of glucocorticoid to the BDNF signaling pathway leading to RISE, since the culture systems is free from the animals’ homeostatic reactions.

In this report, as an application of the present *in vitro* model system to stress research, we investigated at which step stress (represented by the glucocorticoid Dex in the *in vitro* system) interferes with the memory consolidation process. Dex suppressed the establishment of RISE by interfering with the dendritic spine dynamics at the ‘increased fluctuation phase’, which precedes the net increase in the spine number. Furthermore, Dex does not interfere with basal spine dynamics. The ‘increased fluctuation phase’ corresponds to the period where the reorganization of the actin cytoskeleton is enhanced owing to the elevated expression of actin-associated protein cofilin and cofilin-phosphorylating kinase^6^. Since phosphorylated cofilin accelerates actin polymerization and unphosphorylated (or dephosphorylated) cofilin accelerates actin depolymerization^18^, the elevated expression of both cofilin and its kinase should result in the expansion of both unphosphorylated and phosphorylated cofilin; this may explain the increase in both spine generation and retraction.

In the literature, however, it has been reported that exogenously applied glucocorticoids did affect the basal spine dynamics in mouse sensory cortex *in vivo*^19^. It is not clear at present whether this discrepancy arose from the difference in brain regions or from the difference in conditions (*in vitro* versus *in vivo*). It is interesting to address this problem by using the organotypic slice culture of cerebral cortex.

In the present study, we applied Dex after cLTP induction, mimicking a situation where acute excess stress is imposed after an experience. Chronic (and/or weak) stress is known to rather facilitate memory formation *in vivo*^20^,^21^. In this respect, the presence of low amounts of cortisol in the culture medium (<0.3 nM) may influence the interpretation of the present results. Although the acute application of a glucocorticoid receptor antagonist did not show any influence on RISE (nor did mineralocorticoid agonist and antagonist), it would be necessary to apply the antagonists chronically to clarify the chronic effects of the presence or absence of ‘contaminating’ corticoids.

Previously, we reported a different type of structural plasticity in hippocampal slice culture: repeated inductions, but not a single induction, of LTD (long-term depression) led to a long-lasting decrease in synaptic strength accompanied by synapses elimination (LOSS, or LTD-repetition-operated synaptic suppression^17^,^22^,^23^,^24^). Since synapse elimination could also contribute to memory formation, we examined the possible influence of Dex to LOSS. Using the experimental protocols equivalent to the experiment described here, we found that Dex did not interfere with LOSS (Supplementary Fig. S4). Although further studies varying the dosage and timing of Dex application are needed to reach a conclusion, this result suggests excess stress suppresses incremental synaptic plasticity but not decremental synaptic plasticity.

## Methods

### Organotypic slice culture of mouse hippocampus

The culture was prepared as previously described^12^,^25^. C57BL/6 J mouse neonates (6–8 days of age) of a single litter delivered by a single dam were used to prepare a single lot of culture. Briefly, the littermate neonates of both sexes were anesthetized and sacrificed. The hippocampi of both sides were isolated. Two-four slices of 400 μm thickness were cut from dorsoventrally central portion of each hippocampus and pooled with those from littermate neonates. Those slices were then placed on polytetrafluoroethylene filter inserts (Millicell CM; Millipore) and culture medium composed of 50% Hanks MEM, 25% Hanks basal salt solution, and 25% heat-inactivated horse serum (all supplied by Gibco) was supplied from the bottom of the filter. The culture was maintained under humidified air in a 34 °C incubator. Under these conditions, the culture becomes mature and stable after 2 weeks of incubation^4^,^26^.

For morphological examination, the 250 μm-thick hippocampal slices were prepared from Thy1-YFP transgenic mouse line H pups (Jackson Laboratory) that express yellow fluorescent protein in a limited number of CA1 pyramidal cells. The culture was prepared and maintained same as above.

### Quantification of glucocorticoid contents in horse serum

The culture medium contained 25% horse serum. Considerable amounts of glucocorticoid in the serum could produce chronic stress-resembling conditions in culture. We therefore quantified glucocorticoid in the sera by EIA kit (Funakoshi/Arbor Assays) for cortisol (the glucocorticoid species of the horse). The mean +SD of 5 lots of horse serum was 1.21 + 0.34 nM. Hence, the concentration of cortisol in the medium containing 25% serum should be lower than 0.3 nM.

### Induction of chemical LTP and production of RISE

Following the protocols described previously^4^, we induced LTP by chemical means due to the need for long-term maintenance of aseptic conditions in culture. Late-phase LTP is required to produce RISE and is inducible by forskolin (FK; Santa Cruz Biotechnology). The experimental timeline is shown in Fig. 1a. At 19 DIV (days *in vitro*), the cultures were treated as below. The filter insert carrying the slices was transferred to a well containing a stimulation medium composed of Hanks balanced salt solution including FK (20 μM) and transferred again to a well containing a new culture medium including no FK 20 min later. This procedure was repeated three times at 6 hr intervals. Thus the slices grown on that insert were treated identically. The control specimens were treated similarly but with a medium including the vehicle (dimethylsulfoxide) alone. All media used were warmed to 34 °C. The day of the three inductions of chemical LTP (cLTP) was designated as post-stimulus (PS) day 0.

### Application of corticoids and corticoid-related drugs

As shown in the timeline (Fig. 1a), dexamethasone (Dex; a relatively specific glucocorticoid receptor agonist; Sigma-Aldrich; 1–100 nM) or aldosterone (Ald; a mineralocorticoid receptor agonist; Sigma-Aldrich 10 nM) was applied for 24 hr beginning 12 hr after the third induction of cLTP. Application of mifepristone/RU38486 (Mif; a glucocorticoid receptor antagonist; Danco Laboratories; 500 nM) or spironolactone (Spi; a mineralocorticoid receptor antagonist; Pfizer; 500 nM) began 15 min before corticoid application; all applications were terminated simultaneously with corticoid. The way of exposure to the drugs was the same as that to FK explained above.

### Morphological examination with confocal microscopy

The dynamics of the CA1 pyramidal neuron dendritic spines were examined using an Olympus laser-scanning confocal microscope (FV-300; with single-photon optics employing a 60× UPlanSApo60 objective) following the protocol described previously^12^. The timeline of examination is shown in Fig. 1d. One day before the RISE-producing stimulus (*i.e.* PS day −1), one of the slices grown on the filter was cut out together with a piece of underlying filter and transferred to an observation chamber fixed on a temperature-regulated stage (Tokai hit; warmed to 34 °C) of the microscope. A segment of the first or second branch of apical dendrite of a pyramidal cell (located in the anteroposteriorly central region of area CA1 and running nearly horizontally to the focal plane for >10 μm) was imaged at Z-steps of 0.75 μm. The culture was returned to the incubator after imaging. The same dendritic segment was imaged at PS day 3, 6, and 10 repeatedly across days. Examinations of control and experimental specimens in a single series of experiment were made using a single lot of culture. The same examination following the same protocol was repeated multiple times using separate lots of culture to confirm reproducibility. The times of repetition of experiment (*i.e.* the number of lots used) were indicated in figure legends.

### Neuronal cell viability

Glucocorticoids may generate cytotoxicity^27^,^28^. The absence of neuronal cell death under the present experimental conditions was confirmed by enumerating neuronal cell numbers in CA1 and CA3 after immunological staining of a neuronal cell marker, NeuN as described previously^5^. Briefly, samples were fixed with 4% paraformaldehyde and treated with an anti-NeuN monoclonal antibody (Millipore; 1/100 dilution) followed by an Alexa 488-conjugated goat anti-mouse IgG (Invitrogen; 1/200 dilution). The samples were then examined on the confocal microscope described above for the central regions of the areas CA1 and CA3. Focal plane for imaging was fixed at the middle of the samples’ thickness.

### Electrophysiology

Extracellular recordings were performed following the previously described protocol^5^. We designed this experiment to obtain an index for the number of functional synapses. At a pre-determined day, one of the slices were cut out together with a piece of underlying filter and transferred to a recording chamber perfused at the rate of 1 mL/min with an oxygenated artificial cerebrospinal fluid composed of [in mM]: 126 NaCl, 5 KCl, 1.25 NaHPO_4_, 2.5 CaCl_2_, 2.0 MgSO_4_, 22 NaHCO_3_, 10 glucose pH7.3. A glass microelectrode for recording was placed at the somatic layer (where postsynaptic potentials should be integrated) in the anteroposteriorly central region of CA1. A tungsten bipolar electrode for stimulation was placed at the somatic layer of the central part of the area CA3. Upon increasing the stimulation current (pulse of 100 μs duration), the amplitude of the recorded field postsynaptic potential (fPSP) showed a gradual increase followed by saturation. Saturation should occur when practically all inputs to the neurons under recording are activated, and thus the saturated value can be used as an index for the number of synapses formed on the neurons under recording. By changing the recording site more than three times, the maximum value of the saturated fPSP amplitude was taken as an index of the synaptic strength of that slice. Measurements for control and experimental specimens were made within a single lot of culture. The same examination following the same protocol was repeated multiple times using separate lots of culture to confirm reproducibility. The times of repetition of experiment (*i.e.* the number of lots of culture used) were indicated in figure legends.

### Statistical analysis

For the comparison of two sample groups a Welch’s paired *t*-test assuming non-identical variance was applied. For the comparison of >3 sample groups ANOVA followed by Bonferroni test was applied. For the comparison between data sets serially measured on the same samples, 2-factor factorial ANOVA followed by Bonferroni test was applied. The number of samples examined is indicated in each figure. Error bars in the figures indicate standard errors of the means. Statistical significance levels are indicated by asterisks beside the thin lines embracing the pairs of interest: * for *P* < 0.05, ** for *P* < 0.01, *** for *P* < 0.001. To avoid confusion, the asterisks are numbered and color-matched with symbols used in the graph. In addition to the asterisks, the absolute *P* values are shown in figure legends such as 2.22E-2 that means 2.22 × 10^−2^ or 0.0222. Sample pairs exhibiting no statistically significant differences were not labelled.

### Animal care

The study was carried out in accordance with the Regulation on Animal Experiments of the Animal Experiments Committee of Osaka University. The protocol was approved by the Committee for Animal Experiments of Osaka University Graduate School of Frontier Biosciences (No. 12–027).

## Additional Information

**How to cite this article**: Saito, S. *et al.* An *in vitro* reproduction of stress-induced memory defects: Effects of corticoids on dendritic spine dynamics. *Sci. Rep.* **6**, 19287; doi: 10.1038/srep19287 (2016).

## Supplementary Material

Supplementary Figures

## References

[b1] Ebbinghaus H. Über das, Gedächtnis. 1885. Translated in English as *Memory: A Contribution to Experimental Psychology*. (Columbia Univ. 1913).

[b2] Gerfen C. R., Rogawski M. A., Sibrey D. R., Skolnik P. & Wray S.(eds) Short Protocols in Neuroscience: Systems and Behavioral Methods. (Wiley, 2006).

[b3] Pavlov I. P. Lektsii o Rabote Bolyshih Polusharij Golovnogo Mozga. 1927. Translated in English as Conditional Reflexes: An Investigation of the Psychological Activity of the Cerebral Cortex. (Oxford Univ. Press 1927).

[b4] Tominaga-Yoshino K., Kondo S., Tamotsu S. & Ogura A. Repetitive activation of protein kinase A induces slow and persistent potentiation associated with synaptogenesis in cultured hippocampus. Neurosci. Res. 44, 357–367 (2002). 12445624 10.1016/s0168-0102(02)00155-4

[b5] Tominaga-Yoshino K., Urakubo T., Okada M., Matsuda H. & Ogura A. Repetitive induction of late-phase LTP produces long-lasting synaptic enhancement accompanied by synaptogenesis in cultured hippocampal slice. Hippocampus 18, 281–293 (2008). 18058822 10.1002/hipo.20391

[b6] Kawaai K. *et al.* Analysis of gene expression changes associated with long-lasting synaptic enhancement in hippocampal slice cultures after repetitive exposure to glutamate. J. Neurosci. Res. 88, 2911–2922 (2010). 20568283 10.1002/jnr.22457

[b7] Oe Y., Tominaga-Yoshino K. & Ogura A. Local establishment of repetitive long-term potentiation-induced synaptic enhancement in cultured hippocampal slice with divided input pathways. J. Neurosci. Res. 89, 1419–1430 (2011). 21557296 10.1002/jnr.22668

[b8] Kim J. J. & Diamond D. M. The stressed hippocampus, synaptic plasticity and lost memories. Nat. Rev. Neurosci. 3, 453–462 (2002). 12042880 10.1038/nrn849

[b9] Wingenfeld K. & Wolf O. T. Stress, memory, and the hippocampus. Front Neurol. Neurosci. 34, 109–120 (2014). 24777135 10.1159/000356423

[b10] Wiegert O., Pu Z., Shor S., Joels M. & Krugers H. Glucocorticoid receptor activation selectively hampers *N*-methyl-D-aspartate receptor dependent hippocampal synaptic plasticity *in vitro*. Neuroscience 135, 403–411 (2005). 16125856 10.1016/j.neuroscience.2005.05.039

[b11] Krugers H. J. *et al.* Corticosterone shifts different forms of synaptic potentiation in opposite directions. Hippocampus 15, 697–703 (2005). 15959917 10.1002/hipo.20092

[b12] Oe Y., Tominaga-Yoshino K., Hasegawa S. & Ogura A. Dendritic spine dynamics in synaptogenesis after repeated LTP inductions: Dependence on pre-existing spine density. Sci. Rep. 3, 1957 (2013). 23739837 10.1038/srep01957 PMC3674431

[b13] Krugers H. J. *et al.* Corticosterone shifts different forms of synaptic potentiation in opposite directions. Hippocampus 15, 697–703 (2005). 15959917 10.1002/hipo.20092

[b14] Maggio N. & Segal M. Striking variations in corticosteroid modulation of long-term potentiation along the septotemporal axis of the hippocampus. J. Neurosci. Res. 27, 5757–5765 (2007). 10.1523/JNEUROSCI.0155-07.2007 PMC6672761 17522319

[b15] Bear M. F., Connors B. W. & Paradiso M. A. Neuroscience: Exploring the brain. 3rd ed. (Lippincott, Williams & Wilkins 2007).

[b16] Numakawa T., Adachi N., Richards M., Chiba S. & Kunugi H. The influence of glucocorticoids on neuronal survival and synaptic function. BioMol. Concepts 3, 495–504 (2012). 25436554 10.1515/bmc-2012-0012

[b17] Sakuragi S., Tominaga-Yoshino K. & Ogura A. Involvement of TrkB- and p75^NTR^-signaling pathways in two contrasting forms of long-lasting synaptic plasticity. Sci. Rep. 3, 3185 (2013). 24212565 10.1038/srep03185 PMC3822391

[b18] Bamburg J. R., McGough A. & Ono S. Putting a new twist on actin: ADF/cofilins modulate actin dynamics. Trends Cell Biol. 9, 364–370 (1999). 10461190 10.1016/s0962-8924(99)01619-0

[b19] Liston C. & Gan, W. B. Glucocorticoids are critical regulators if dendritic spine development and plasticity in vivo. Proc. Natl. Acad. Sci. USA 108, 16074–16709 (2011). 10.1073/pnas.1110444108 PMC3179117 21911374

[b20] Diamond D. M., Bennett M. C., Fleshner M. & Rose G. M. Inverted-U relationship between the level of peripheral corticosterone and the magnitude of hippocampal primed burst potentiation. Hippocampus 2, 421–430 (1992). 1308198 10.1002/hipo.450020409

[b21] Conrad C. D. A critical review of chronic stress effects on spatial learning and memory. Prog. Neuropsychopharmacol. Biol. Psychiatry 34, 742–755 (2010). 19903505 10.1016/j.pnpbp.2009.11.003

[b22] Shinoda Y., Kamikubo Y., Egashira Y., Tominaga-Yoshino K. & Ogura A. Repetition of mGluR-dependent LTD causes slowly developing persistent reduction in synaptic strength accompanied by synapse elimination. Brain Res. 1042, 99–107 (2005). 15823258 10.1016/j.brainres.2005.02.028

[b23] Kamikubo Y. *et al.* Long-lasting synaptic loss after repeated induction of LTD: Independence to the means of LTD induction. Eur. J. Neurosci. 24, 1606–1616 (2006). 17004924 10.1111/j.1460-9568.2006.05032.x

[b24] Hasegawa S., Sakuragi S., Tominaga-Yoshino K. & Ogura A. Dendritic spine dynamics leading to spine elimination after repeated inductions of LTD. Sci. Rep. 5, 7707 (2015). 25573377 10.1038/srep07707 PMC4648349

[b25] Stoppini L., Buchs P. A. & Muller D. A simple method for organotypic cultures of nerve tissue. J. Neurosci. Meth. 37, 173–182 (1991). 10.1016/0165-0270(91)90128-m 1715499

[b26] Muller D., Buchs P. A. & Stoppini L. Time course of synaptic development in hippocampal organotypic culture. Brain Res. 71, 93–100 (1993). 10.1016/0165-3806(93)90109-n 8432004

[b27] Sapolsky R. M. The physiological relevance of glucocorticoid enhdangerment of the hippocampus. Ann. N. Y. Acad. Sci. 746, 294–304 (1994). 7825884 10.1111/j.1749-6632.1994.tb39247.x

[b28] Rogalska J. Mineralocorticoid and glucocorticoid receptors in hippocampus: Their impact on neurons survival and behavioral impairment after neonatal brain injury. Vitamin Horm. 82, 391–419 (2010). 10.1016/S0083-6729(10)82020-5 20472149

