# Supplementary material for: An in vitro reproduction of stress-induced memory defects: Effects of corticoids on dendritic spine dynamics
Source: Sci Rep. 2016 Jan 14;6:19287. doi: 10.1038/srep19287 (PMC4725889; doi:10.1038/srep19287)
Supplement: Supplementary Figures [file srep19287-s1.pdf]

Supplementary information to:

"An *in vitro* reproduction of stress-induced memory defects: Effects of corticoids on dendritic spine dynamics"

by Saito, Kimura, Adachi, Numakawa, Ogura & Tominaga-Yoshino

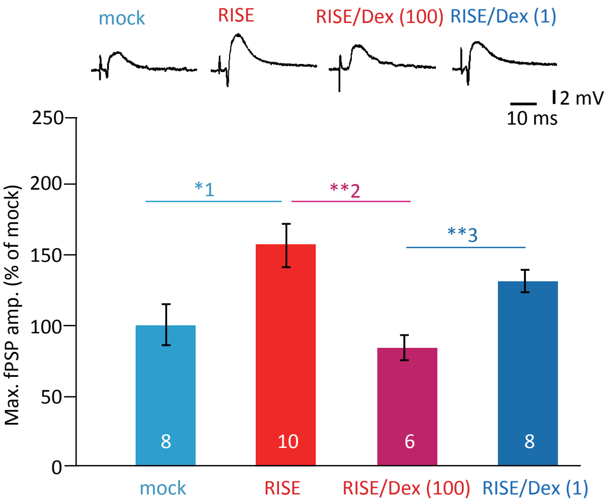

Supplementary Figure S1| Dose-effect relationship of Dex on suppression of RISE. The suppression seen from functional index (fPSP measured at PS day 14) is depicted here. For the morphological index, see Fig. 2 in the main text. As explained in Methods section of the main text, the control (mock) and experimental samples were subjected to fPSP measurements on the same day using a single lot of culture. The mean value of control samples was taken as 100% for each lot. The same measurement was repeated 4 times using 4 lots of culture (*i.e.* 4 dams). Absolute *P* values are 2.18E-2 for *1, 4.73E-3 for **2, 1.96E-3 for **3.

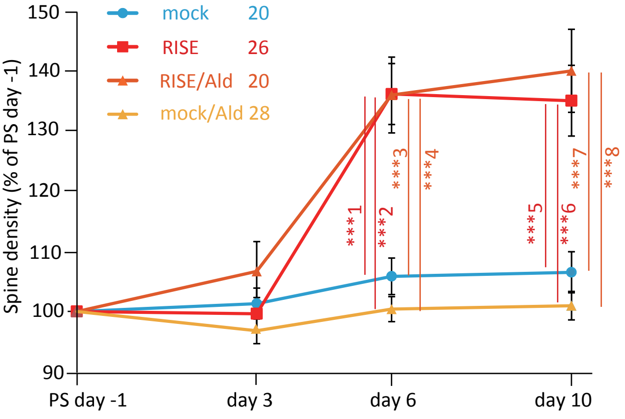

Supplementary Figure S2|Absence of RISE-suppressive effect of mineralocorticoid. Aldosterone (Ald, 10 nM) was applied for 24 hr beginning 12 hr after the third induction of cLTP. The morphological index (time course of spine density changes) is depicted here. For the functional index, see Fig. 3 in the main text. As explained in Methods section of the main text, the control (mock) and experimental samples were subjected to spine density measurements on the same day using a single lot of culture. The mean value of control samples was taken as 100% for each lot. The same measurement was repeated 4 times using 4 lots of culture (*i.e.* 4 dams). Absolute *P* values are 2.29E-5 for ***1, 5.25E-8 for ***2, 8.09E-5 for ***3, 4.22E-7 for ***4, 5.41E-4 for ***5, 4.01E-6 for ***6, 1.09E-4 for ***7, 8.94E-7 for ***8.

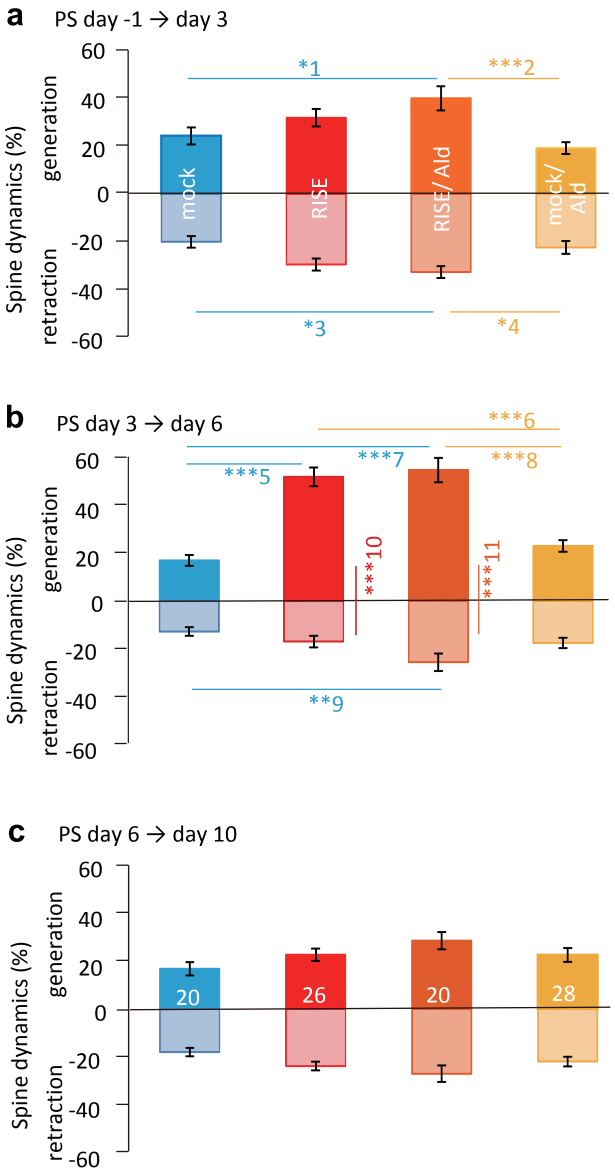

Supplementary Figure S3| Dendritic spine dynamics in RISE, and no interference by Ald. The methods are the same as the case of Dex, except for the steroid species applied (see Fig. 4 in the main text). The same spine fate chasing was repeated 4 times using 4 lots of culture (*i.e.* 4 dams). Absolute *P* values are 3.52E-2 for *1, 6.90E-4 for ***2, 1.20E-2 for *3, 3.90E-2 for *4, 4.81E-9 for ***5, 9.78E-8 for ***6, 2.73E-9 for ***7, 5.20E-8 for ***8, 8.89E-3 for **9, 7.11E-10 (Welch´s *t*-test) for ***10, 3.85E-5 (Welch´s *t*-test) for ***11.

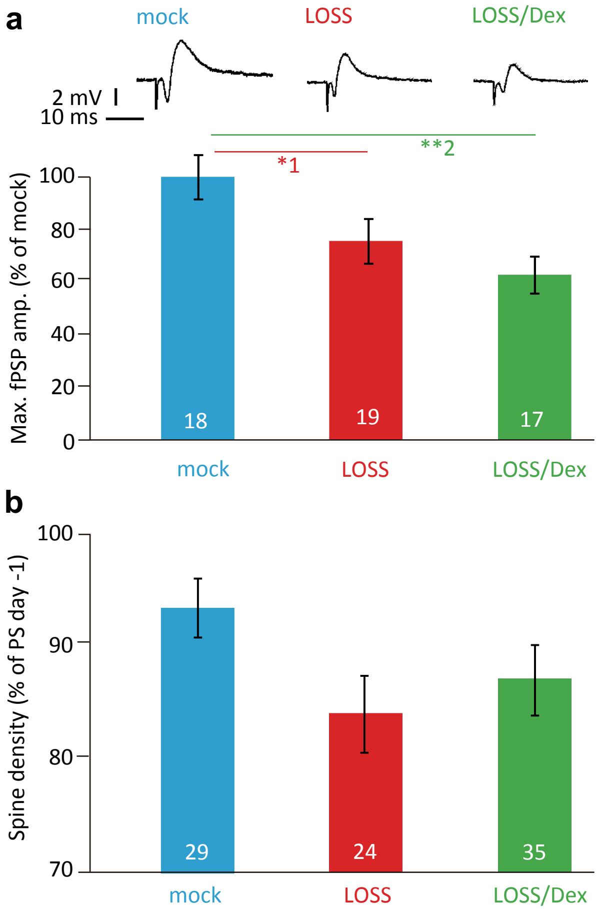

Supplementary Figure S4 | Absence of interference of Dex with LOSS. LOSS, or LTD-repetition-operated synaptic suppression (a decremental form of long-lasting synaptic plasticity coupled with synapse elimination in hippocampal slice culture), was produced by three repeated inductions of LTD by means of DHPG (dihydroxyphenylglycine, an mGluR agonist, 50 µM, 10 min, 3 times at 6 hr intervals) application, (according to the same protocol as used for the production of RISE by FK application described in the main text). (a) The functional index (maximal fPSP amplitude at PS day 14) was compared. Note that the synaptic strength was significantly reduced in 3x cLTD (LOSS) samples, but Dex did not suppress the reduction. The same fPSP measurement was repeated 6 times using 6 lots of culture (*i.e.* 6 dams). Absolute *P* values are 4.08E-2 for *1, 2.00E-3 for **2. (b) The morphological index (spine density at PS day 10) was compared. Note that the spine density showed decreasing tendency in 3x cLTD (LOSS) samples, but Dex did not suppress the tendency. The same spine density measurement was repeated 4 times using 4 lots of culture (*i.e.* 4 dams).
